# The effect of implementing mind maps for online learning and assessment on students during COVID-19 pandemic: a cross sectional study

**DOI:** 10.1186/s12909-022-03211-2

**Published:** 2022-03-12

**Authors:** Amany A. Alsuraihi

**Affiliations:** Physics Department, Faculty of Science, King Abdulaziz University, Jeddah, 21589 Saudi Arabia

**Keywords:** COVID-19 pandemic, Medical education, Mind mapping, Online assessment, Online learning, Online education

## Abstract

**Background:**

In Saudi Arabia, the sudden shift from conventional (in-person) to online education due to the coronavirus disease 2019 (COVID-19) pandemic affected teaching and assessment methods. This research aimed to assess the effectiveness of mind maps in this regard, measure students’ reactions to certain educational environment-related changes caused by the pandemic, and identify skills that students perceived they gained through mind mapping.

**Methods:**

This study employed a non-intervention (cross sectional) design. Participants were King Abdulaziz University students from two medical physics courses (second and fourth level). Data were collected twice (after the first and last mind mapping assignments), and responses were analyzed using a paired t-test. Overall student results were compared against overall student performance in the previous term using chi-squares test hypothesis testing. The data were collected and analyzed using SPSS software.

**Results:**

The results of the paired t-test showed no significant differences between students’ mean satisfaction in both surveys. Nevertheless, students’ responses revealed their satisfaction with using mind maps. Moreover, students believed that they gained skills like organizing and planning, decision making, and critical thinking from the mind map assignments. The chi-squares test (Chi-square = 4.29 < ${x}_{\mathrm{0.05,4}}^{2}$  = 9.48 and *p*-value = 0.36 > 0.05) showed no differences in students’ grade distribution between the two terms of 2020 (pre- and post-COVID-19 pandemic) despite the change in assessment style post-pandemic commencement.

**Conclusions:**

Mind mapping can be adapted as an online teaching and assessment method. Additionally, student support and education institution-level effective communication can reduce stress during challenging times.

**Supplementary Information:**

The online version contains supplementary material available at 10.1186/s12909-022-03211-2.

## Background

On January 7, 2020, coronavirus disease 2019 (COVID-19) was identified as the cause of a cluster of pneumonia cases in Wuhan, China [1]. Since then, millions of people globally have been infected with COVID-19, and many families have been affected. To protect against the rapid spread of the virus, the World Health Organization recommends that people should maintain social distancing, wear masks, and practice good hygiene at all times. On March 8, 2020, following these recommendations, the government of Saudi Arabia closed educational institutions, including universities and schools at all levels, to control the spread of COVID-19 and protect the population. Hence, to maintain the safety of students and school staff while continuing to provide education, the Saudi Ministry of Education [2] made a sudden switch from conventional in-person education to virtual online education via different platforms. For instance, King Abdulaziz University began using the Blackboard platform. Support including workshops, necessary learning materials, and helplines were provided to ensure a smooth transition. However, many exams and quizzes were cancelled, and lecturers were directed to assess students' progress using other methods.

In the education field, and specifically in medical education, many learning strategies and tools have been designed to enhance students’ learning experiences, such as case-based teaching [3], role playing [4], problem-based learning, discussion groups [5], didactic learning [6], concept maps [7], and mind maps [8]. Both concept maps and mind maps are excellent learning tools to enhance students’ abilities to formulate concepts, analyze data, as well as connect ideas and understand the relationships between them. They both involve visual knowledge reconstruction, are easier to follow and engage with than verbal and written scripts, and provide an interactive learning method for students. However, they have certain differences. Concept mapping has a top-down structure that begins with the main concept/idea and branches out into sub-concepts/ideas, which are enclosed in circles or boxes. Association phrases and arrows are used to demonstrate the relationship between them. The structure could be hierarchical, non-hierarchical or data-driven. Some of the drawbacks of using concept maps are their limited extensibility, low memorability due to the level complexity of certain concepts, and the medium to high level of difficulty, as it requires expertise and training to master concept maps [9, 10].However, mind maps are easier to grasp, encourage creativity, enhance engagement, promote ownership of ideas, and involve both brain hemisphere [9]. While Eppler’s study [9] shows that combining more than one visual mapping method can be of more benefit than using individual techniques, Martin Davies [10], who studied both concept maps and mind maps, claims that the choice of mapping technique depends predominantly on the aim and purpose of its use. Therefore, in the present study, mind mapping was chosen as an online learning and group assessment tool in two medical physics courses during the sudden changes brought about by the pandemic. This occurred approximately during the mid-second term, Spring 2020.

## Literature Review

The mind mapping technique was developed by Buzan [11], based on a theory inspired by da Vinci’s notes, and by scientists such as Galileo, ﻿Feynman, and Einstein. A mind map starts with the main topic represented as an image at the center of the page, then the subtopics branch out and are placed onto curved lines. A keyword or image is included on each branch to represent ideas. These ideas continue to branch out as far as is required by the subject. Furthermore, connection lines, codes, and symbols are used to connect ideas, highlight important concepts, and stimulate creative thinking [11, 12]. Many studies have explored the benefits of Buzan’s technique for teaching and learning, such as retaining information, organizing thoughts, and developing critical-thinking skills (e.g., reasoning, decision-making, and problem-solving) [11, 12]. In a study conducted with elementary school teachers [13], teachers agreed that using mind maps in education would stimulate students’ creativity, enhance learning and memorization, and serve as a tool to assess students’ degree of comprehension for the topics being taught. Moreover, Ellozy and Mostafa [14] studied the feasibility of using a hybrid concept comprising mind mapping strategies among first-year students at the American University in Cairo and found that their technique enhanced students’ critical-thinking and reading comprehension skills, ability to engage in visualization, and imagination during learning and communicating ideas. Further, it was useful as an assessment tool for teachers to evaluate students’ systematic thinking [14]. Wu and Wu [15] showed that using mind mapping in medical education improved nursing students’ critical-thinking abilities and ﻿stimulated their eagerness to learn during their internship. This supported the findings of a previous study [8] that investigated the effectiveness of mind mapping as an active educational tool in a﻿ Nursing Management course to enhance students’ critical-thinking skills; the students’ scores were found to have improved. Mind maps were named as a factor contributing to the high-achieving medical students’ educational success [16]. Mind maps have also been used to enhance health education of patients and improve psychological well-being in cancer patients [17]. Chen [17] developed a mind map-based life review program (MBLRP), which is conducted through several sessions for the life review aspect (from childhood to adulthood, their cancer experience, and then a summary session of the life experience). Sessions use mind maps, videos and photos. Results show that the MBLRP is a promising intervention to promote psychological wellbeing among patients, while being enjoyable, feasible, and easily accepted. Another study done by Tan et al. [18] concluded that mind mapping can improve the effectiveness of health education and guidance for patients with lung cancer who were undergoing chemotherapy, where the level of perceived control improved symptom distress; the longer the period of health education, the better the effect. Yang et al. [19] investigated the effectiveness of using mind mapping as a health education tool for children with cavities who were in extended care, as well as their parents. The results show an increase in child and parent compliance with health education, which is evident from an increase in cavity knowledge and more follow-up visits to the dentist. Mind map have also proven effective as a language learning material for students; for example, Petrova and Kazarova [20] discussed the feasibility of using mind maps as a teaching and learning tool in foreign language acquisition, as it helps students to be independent learners and solve diverse problems. Kalizhanova et al. [21] created a trilingual e-dictionary using a mind map program for high school students, to teach them biological terms in Kazakhstan. Furthermore, Alahmadi’s [22] study showed a significant improvement in vocabulary acquisition by students who used mind maps as a learning strategy for English language vocabulary as a second language. Mind maps were also used in science and engineering as educational material, learning exercises, and a critical thinking tool for collaborative and independent learning. Gagic et al. [23] used mind maps to teach physics to primary school students; results showed an increase in student achievement and a decrease in the mental effort necessary to study physics, when compared to conventional teaching and learning methods. In the teacher education program for mathematics, Araujo and Gadanidis [24] have developed a theory to promote mathematical and pedological knowledge construction by applying an online collaborative mind mapping exercise to two educational courses: computational thinking in mathematics education and mathematics teaching methods. In a study conducted by Allen et al. [25], mind maps were used in flipped learning activities in a chemistry lab, which included students with special needs. Students were reflective on their learning, collaborative with their peers, and engaged with each other during the activity. This enhanced their critical thinking, deepened their knowledge, and strengthened their interpersonal skills. In the field of engineering, Lai and Lee [26] show that mathematical engineering students’ achievement levels increased by using mind maps, while their cognitive load reduced. Hence, using effective learning and teaching techniques like mind maps reduces students' cognitive load. Chen et al. [27] have studied the collaborative behavior of engineering students while doing brainstorming activities using mind maps. Their study analyzed the change in students’ behavior during mind map tasks and issues that arose to implement in the design of digital mind map tools. Selvi and Chandramohan [28] have used mind mapping technique in collaborative tasks for a mechanical engineering course, which increased students' academic achievement and motivation to learn. Here, using mind maps in collaborative and interactive learning settings enhanced students’ ability to recall technical terms. To the author’s knowledge, mind maps have not yet been investigated in a medical physics course. However, previous studies done on mind maps for medical, science, and engineering courses showed that they are on the same level as medical physics courses in terms of their cognitive load. Hence, it should be a suitable learning and assessment tool during the pandemic. Therefore, this study aims to assess the effectiveness of mind mapping as a learning and assessment tool for medical physics students, while under stress from the COVID-19 pandemic and while experiencing the related educational mode changes. Accordingly, the following were assessed:

- Student satisfaction with the provided information on mind mapping and associated course assignments.
- Effects of changing the assessment style due to switching from on campus to online classes in relation to students’ satisfaction.
- Student satisfaction with using mind maps as a learning tool and student perceptions of the skills gained from the assignment.

To evaluate these objectives, this study developed two online surveys (Surveys 1 and 2) It hypothesized that students could improve satisfaction by practicing mind maps and that academic achievement would be unaffected by the pandemic compared to previous pre-pandemic commencement results. This study could set the basis for future studies when adapting mind maps as an online learning and assessment method and presenting a measure for students’ satisfaction and perception on the technique.

## Methods

During the online teaching period from March through the end of April 2020, three mind map assignments, one set of critique questions, and two surveys were given to students in two different courses as an alternative teaching and assessment method. These three assignments covered important topics in both courses, as the grading system was changed by the Ministry of Education due to the COVID-19 pandemic. Therefore, mind mapping assignments were chosen as an alternative, to enhance students’ learning and memorizing and for use as an assessment tool. Performing this assignment as part of a group in such sudden and stressful circumstances could help students acquire soft skills, such as conflict resolution and time management. Moreover, results of total students’ achievement in these courses in the second term were compared with students’ achievement level in the first term of 2020, prior to the COVID-19 outbreak, using hypothesis testing.

The sample chosen for the study was the cluster sampling from the Kingdom of Saudi Arabia, Jeddah City, King Abdulaziz University, Department of Physics, Female Campus. Participants were medical physics students. The sample includes only female students, as the female and male campuses are separated at Saudi Arabian Universities. The study was conducted with female students enrolled in two separate medical physics courses taught by the author of the study: 1) health physics, a second-level introductory course, and 2) magnetic resonance and medical imaging (MRI), taught to fourth-year students. The sample of the study was 55 students in total, from both courses. Students were verbally informed that 1) survey participation would be voluntary, 2) responses would be anonymous, and 3) non-participation would not affect their course grades, as no identifying information would be collected. Thus, participation was considered to imply consent.

### Assignments

To perform the mind mapping assignments, students were first assigned randomly to groups of 3–6 students using the Blackboard system. There were 11 students in the MRI course; thus, students were divided into 3 groups. There were two classes for the health physics course, one with 14 students and one with 30 students, who were divided into 3 and 5 groups, respectively. Thus, 55 students in total were given three mind map assignments that covered the most important course topics. Each mind map covered a section of the course that students had already completed.

All mind mapping assignments were posted on Blackboard with instructions for guidance. Students also participated in a short session in which the assignments were explained, with an emphasis on the resources provided to assist them and the grading style (rubric). Students were directed to engage in self-learning on the Blackboard platform by using the different posted YouTube video instructions and resources in Arabic and English on how to create a mind map. Students were also given the freedom to choose and download one of the three online mind mapping software apps: MindMaster [29], MindMeister [30], and XMind [31]. Online materials on how to use each tool were also posted for students on Blackboard.

Based on concept map assessment criteria published online, the author designed a mind map rubric to assess students’ accomplishments (see Appendix A in Additional File 1; [32, 33]). After each mind mapping assignment, students received feedback on their work on Blackboard based on the rubric.

After these assignments were completed, a fourth assignment was posted for students on Blackboard. This assignment included mind maps that the students had worked on in each course: 23 for the health physics course, 8 for the MRI course, and a set of 3 questions. The mind maps with the highest scores were excluded from this assignment. Students were directed to choose a mind map that they did not work on and answer the three critique questions. The questions were as follows: “Does this mind map include all ideas and concepts related to the subject? If not, state what is missing? State only one aspect that is missing;” “What are the aspects that you like most about the mind map (e.g., in terms of ideas, links and connections used, supporting evidence, information used);” and “Provide at least one suggestion to improve this mind map.” The motivation behind this assignment was to encourage students to constructively critique their peers’ work. Therefore, there were no wrong answers to 2 out of the 3 questions, unless students failed to spot their peers’ mistakes regarding missing information on the mind map. The results of this assignment are not shown in this paper.

### Surveys

To address study objectives, a survey was distributed online to students in both courses after the first assignment (Survey 1), Appendix B in Additional File 1. It was divided into three main sections: student satisfaction with the information regarding mind mapping and associated assignments; effects on students regarding the change in assessment style due to COVID-19 control measures and the transition to online education; and student satisfaction with using mind maps as a learning tool and the skills gained from the assignment. Responses to the questions in each section were rated on a five-point Likert scale ranging from “strongly agree” to “strongly disagree.” Additionally, an extra question on the skills students believed they acquired when working on their assignments was included.

After submitting the last assignment, the same survey was posted for students again with an extra open-ended question: “After finishing all three mind mapping assignments, what do you think are the positive and negative aspects of the assignments?” “Do you have any suggestions about them?” (Survey 2). This was done to compare students’ responses from both surveys and measure changes in their perceptions regarding mind mapping through their openly shared views on the assignments.

### Statistical analysis

To address the research questions, students were asked to complete a survey after the first assignment and again after the last. The data collected from students for first survey S1 is available in Additional file 2 while the data collected from the second survey S2 is available in Additional file 3. Students’ responses to the two surveys were then compared, as were their achievement levels in the first term before the COVID-19 pandemic and the second term after the pandemic began. The data for students’ academic achievement in first and second term can be found in Additional file 4. Both Survey 1 (S1; N = 53 students) and Survey 2 (S2; N = 45 students) had one independent variable—medical physics students (health physics and MRI). However, the dependent variable for S1 represented students’ answers related to the survey’s objectives, while for S2, they represented students’ answers related to the survey’s objectives and to an open-ended question. To measure the internal consistency of the survey items, Cronbach’s Alpha was used. It creates a measure of reliability for the survey items and how closely related a set of items within a group are. Factor analysis was used to test the validity between the survey questions in subsites. If the value is less than the absolute value of 0.4, it is inconsistent and saturated. This analyses the relationships between the set of survey questions that are grouped per survey aim (subset), to determine whether the participant’s responses on different questions per survey aim (subsets) relate more closely to one another than to other survey questions [34].

Responses to positively worded questions were collected and coded as follows: strongly agree = 5, agree = 4, neutral = 3, disagree = 2, and strongly disagree = 1. However, to maintain consistency, for Questions 1 and 2 in the second section of the survey (Appendix B in Additional File 1), the negatively worded questions were taken into consideration, and responses to these were reverse coded. The survey data were analyzed using the SPSS software package. Frequencies (N), percentages (%), means (M), and standard deviations (SD) were used to analyze the response. Responses to the open-ended question from S2 were low (only 19 response) and hence not statistically significant. Therefore, the coding technique was used, and similar answers were added into the same category. Answers were categorized as positive, negative, and in the form of suggestions for mind mapping assignments.

The means of both surveys' first and second objectives were tested for their significance of difference using a paired samples t-test. Finally, a chi-squared test was used to determine any statistically significant differences between student achievement levels in the first term (pre-COVID-19 pandemic) and the second term (post-COVID-19 pandemic commencement).

## Results

Responses from the questionnaires of S1 and S2—which were provided prior to the final exams after students submitted all assignments and were given feedback on their work and scores based on the rubric—were analyzed. Cronbach’s alpha was 0.829 and 0.775 for S1 and S2, respectively, indicating a relatively high internal consistency for both surveys. Table 1 show the result of factor analysis for both surveys S1 and S2. Results show good internal validity values, except for Question 6 in S1, while showing good internal validity for S2. This could be due to the sample size, as the larger the sample size, the better the statistical power, which reflects on factor analysis values [34]. As shown in Table 2, the highest responses for S1 and S2 were from the health physics course, which made up 81% and 80% of the responses, respectively. The remaining 19% and 20% responses in S1 and S2, respectively, were from the MRI course students. This similarity in results was expected, as more students were registered for the health physics course.

**Table 1:** Factor analysis with principle component analysis for Survey 1 and 2

SURVEY 1							SURVEY 2					
Objective 1		Objective 2			Objective 3		Objective 1		Objective 2		Objective 3	
Items	Component 1	Items	Component 1	Component 2	Items	Component 1	Items	Component 1	Items	Component 1	Items	Component 1
Q1	0.425	Q5	0.790	0.412	Q8	0.858	Q1	0.546	Q5	0.871	Q8	0.933
Q2	0.919	Q6	0.869	-0.050	Q9	0.892	Q2	0.685	Q6	0.883	Q9	0.872
Q3	0.805	Q7	-0.305	0.924	Q10	0.882	Q3	0.829	Q7	-0.410	Q10	0.775
Q4	0.732				Q11	0.529	Q4	0.751			Q11	0.745
					Q12	0.831					Q12	0.496

**Table 2:** Number of responses in survey 1 and 2

Course	S1 [N (%)]	S2 [N (%)]
MRI – JAR	10 (18.9%)	9 (20%)
HP – GAR	31 (58.5%)	23 (51.1%)
HP – IAR	12 (22.6%)	13 (28.9%)
Total	53 (100%)	45 (100%)

S1: Survey 1, S2: Survey 2, MRI – JAR: Magnetic Resonance and Medical imaging course, course code: JAR, HP: Health Physics Course, course’s codes: GAR and IAR

Table 3 provides detailed information on the mean, standard deviation, and trend for responses to each question regarding student satisfaction with the information provided to them about mind mapping and the associated course assignments in S1 and S2. The overall mean frequency for responses in S1 and S2 was 3.38 and 3.53, with a trend of “neutral” and “agree” responses, respectively. Thus, students indicated overall satisfaction with the information provided about mind mapping and the associated course assignments in S2. Additionally, S2 results showed that 60% of students had not been previously trained on mind mapping (Q1), while 26.7% of students had used mind maps in their studies before. Regarding the usefulness of the supporting information provided (Q2) and recommended instructional videos (Q3), 66.7% and 57.8% of students found this to be useful, respectively, while only approximately 4% and 18% did not, respectively. Most students (73.4%) agreed that the assessment method (rubric) the instructor provided was clear (Q4), while 13.4% did not understand the assessment method.

**Table 3:** Student satisfaction with information provided about mind mapping and assignments

Questions	Survey	Strongly disagree		Disagree	Neutral	Agree	Strongly agree	Mean	Standard deviation	Trend
		N (%)								
1- I have learned about and used mind maps in my studies before	S1	22 (41.5%)		11 (20.8%)	10 (18.9%)	8 (15.1%)	2 (3.8%)	2.19	1.241	Disagree
	S2	17 (37.8%)		10 (22.2%)	6 (13.3%)	3 (6.7%)	9 (20%)	2.49	1.547	
2-The information the instructor provided on Blackboard about how to create mind maps was very useful to me	S1	2 (3.8%)		6 (11.3%)	17 (32.1%)	13 (24.5%)	15 (28.3%)	3.62	1.130	Agree
	S2	1 (2.2%)		1 (2.2%)	13 (28.9%)	13 (28.9%)	17 (37.8%)	3.98	0.988	
3-The information the instructor provided on Blackboard about mind mapping software was very useful to me	S1	3 (5.7%)		9 (17%)	12 (22.6%)	14 (26.4%)	15 (28.3%)	3.55	1.234	Agree
	S2	4 (8.9%)		4 (8.9%)	11 (24.4%)	10 (22.2%)	16 (35.6%)	3.67	1.297	
4-The information the instructor provided about grading the mind maps was clear	S1	1 (1.9%)		5 (9.4%)	6 (11.3%)	14 (26.4%)	27 (50.9%)	4.15	1.08	Agree
	S2	3 (6.7%)		3 (6.7%)	6 (13.3%)	12 (26.7%)	21 (46.7%)	4.00	1.225	
	S1		Mean for this goal					3.38		Neutral
	S2							3.53		Agree

S1: Survey 1; S2: Survey 2

Table 4 provides detailed information on the responses to each question regarding the effects of changing the assessment style due to COVID-19 control measures and switching from on-campus classes to online virtual classes and students’ satisfaction levels. S1 and S2 results show a mean score of 3.06 and 2.98, respectively, indicating a generally neutral response.

**Table 4:** The effect of changing assessment styles due to COVID-19 spread control measures and the transition to online education on students’ satisfaction

Questions	Survey	Strongly disagree		Disagree	Neutral	Agree	Strongly agree	Mean	Standard deviation	Trend
		N (%)								
1- Switching course assessment methods in general from paper quizzes and tests to online assignments was not stressful* to me	S1	21 (39.6%)		11 (20.8%)	9 (17%)	5 (9.4%)	7 (13.2%)	2.35	1.429	Disagree
	S2	19 (42.2%)		10 (22.2%)	5 (11.1%)	2 (4.4%)	9 (20%)	2.37	1.556	Disagree
2- Switching the assessment method in this course from paper quizzes and tests to online assignments, mind mapping projects and online MCQ tests was not stressful* to me	S1	11 (20.8%)		8 (15.1%)	14 (26.4%)	11 (20.8%)	9 (17%)	2.98	1.380	Neutral
	S2	12 (26.7%)		7 (15.6%)	14 (31.1%)	2 (4.4%)	10 (22.2%)	2.80	1.471	Neutral
3- I am satisfied with the changes in grade distribution	S1	3 (5.7%)		4 (7.5%)	11 (20.8%)	15 (28.3%)	20 (37.7%)	3.85	1.183	Agree
	S2	1 (2.2%)		3 (6.7%)	17 (37.8%)	8 (17.8%)	16 (35.6%)	3.78	1.085	Agree
	S1		Mean for this goal					3.06		Neutral
	S2							2.98		Neutral

^*^ To maintain consistency with analysis as reverse coding in Likert scale for Q1, Q2 was used with negative wording ("not stressful" here). S1: Survey 1; S2: Survey 2

As shown in Table 5, student satisfaction with using mind maps as a learning tool and the skills they gained from the assignments had a mean frequency of 3.75 and 3.56 in S1 and S2, respectively, with a general trend of “agree” responses; this reflects a consensus among students regarding their satisfaction with using mind maps as a learning tool. In S1 and S2, approximately 64% and 53.4% of the students agreed that using mind maps was beneficial to their learning in and understanding, while only 15% and 9% believed it was not, respectively (Q1). Furthermore, 69% of students agreed that working on the mind maps helped them recognize and identify themes in the lectures, while 9% did not in both S1 and S2 (Q2). In addition, when students were asked if using mind maps helped them gain a deeper understanding of the subject they were studying, 59% in S1 and 58% in S2 agreed that it was beneficial to them, while 21% in S1 and 18% in S2 did not find it useful (Q3). Regarding working on the mind mapping assignment in groups, 51% and 42% of students agreed they had gained new skills, compared to 26% and 31% who were not satisfied with their group in S1 and S2, respectively (Q4). However, when students were asked if they would continue using mind maps in their studies in S1 and S2, 68% and 60% stated they would, while only 19% and 16% reported they would not, respectively (Q5).

**Table 5:** Students’ satisfaction with using mind maps as a learning tool

Questions	Survey	Strongly disagree		Disagree	Neutral	Agree	Strongly agree	Mean	Standard deviation	Trend
		N (%)								
1- Using mind maps was beneficial to my learning in and understanding of the course	S1	1 (1.90%)		7 (13.20%)	11 (20.80%)	16 (30.20%)	18 (34%)	3.81	1.11	Agree
	S2	1 (2.20%)		3 (6.70%)	17 (37.80%)	16 (35.60%)	8 (17.80%)	3.6	0.939	Agree
2- Using mind maps helped me identify the main ideas in the lectures	S1	0 (0%)		5 (9.40%)	7 (13.20%)	18 (34%)	23 (34.40%)	4.11	0.974	Agree
	S2	1 (2.20%)		3 (6.70%)	10 (22.20%)	17 (37.80%)	14 (31.10%)	3.89	1.005	Agree
3- Using mind maps helped me to understand the subject in depth	S1	1 (1.90%)		10 (18.90%)	11 (20.80%)	14 (26.40%)	17 (32.10%)	3.68	1.173	Agree
	S2	3 (6.70%)		5 (11.10%)	11 (24.40%)	15 (33.30%)	11 (24.40%)	3.58	1.177	Agree
4- I gained new skills working in a team for the mind mapping projects	S1	10 (18.90%)		4 (7.50%)	12 (22.60%)	12 (22.60%)	15 (28.30%)	3.34	1.454	Neutral
	S2	6 (13.30%)		8 (17.80%)	12 (26.70%)	13 (28.90%)	6 (13.30%)	3.11	1.247	Neutral
5- I will continue to use mind maps in my future courses	S1	5 (9.40%)		5 (9.40%)	7 (13.20%)	15 (28.30%)	21 (39.60%)	3.79	1.321	Agree
	S2	3 (6.70%)		4 (8.90%)	11 (24.40%)	14 (31.10%)	13 (28.90%)	3.67	1.187	Agree
	S1		Mean for this goal					3.75		Agree
	S2							3.56		Agree

S1: Survey 1; S2: Survey 2

**Fig. 1:**
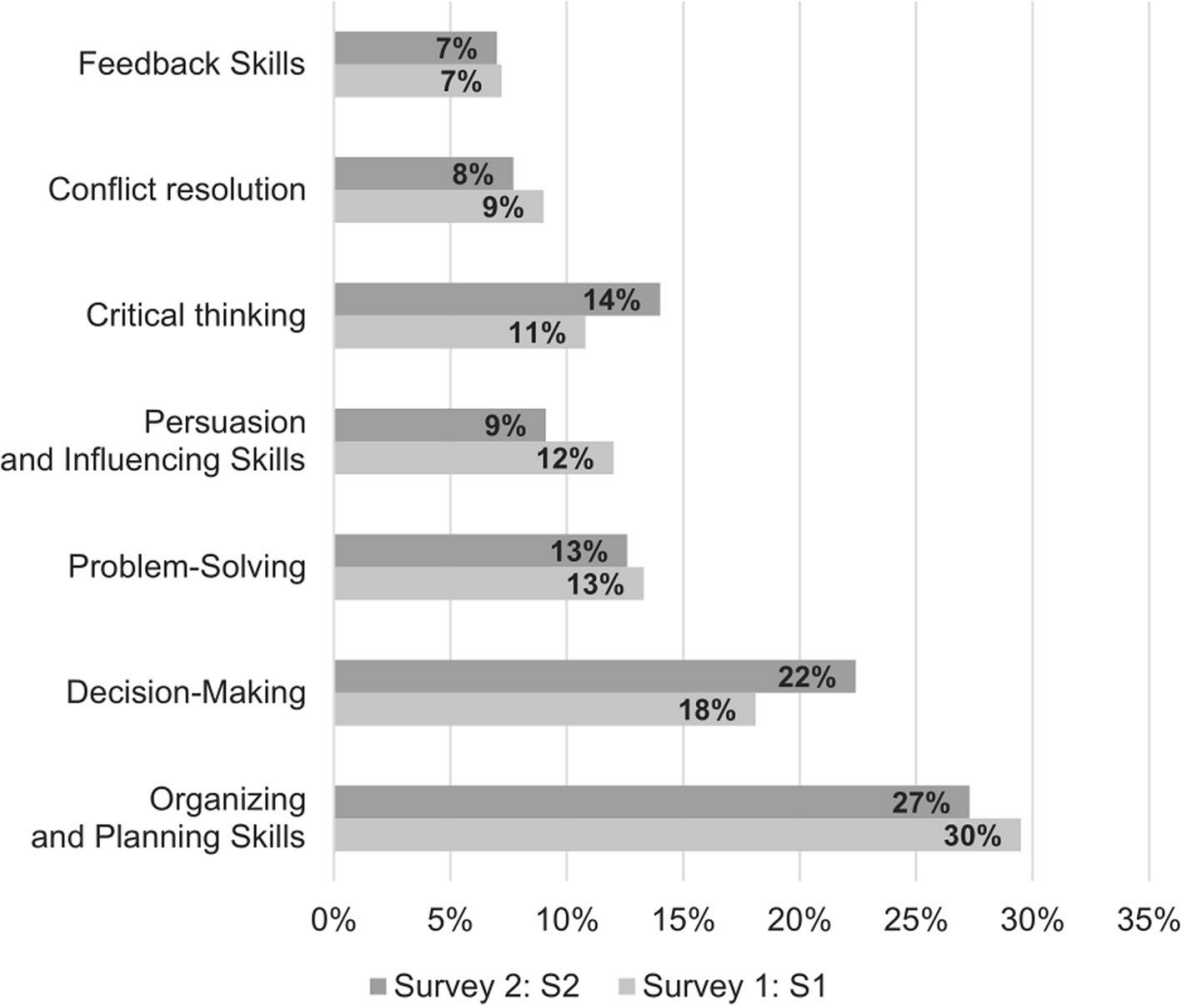
Bar chart representing students’ perceptions in Survey 1 and 2 regarding each skill gained during their group work on the mind mapping assignment

Figure 1 summarize students’ responses in S1 and S2, regarding the skills they perceived themselves as gaining by participating in the mind mapping assignments. Student responses in both surveys suggested that the top skill gained was organizing and planning (S1: 30%; S2: 27%), followed by decision-making skills (S1: 18%; S2: 22%). However, in S1, the proportions of students who gained problem-solving (13%) and persuasion and influencing skills (12%) were almost equal, followed by 11% of students gaining critical thinking skills. Conversely, in S2, nearly an equal proportion of students perceived gaining critical-thinking (14%) and problem-solving (13%), followed by 9% of students gaining persuasion and influencing skills. Finally, student responses in both surveys reported that conflict-resolution (S1: 9%; S2: 8%) and feedback (S1: 7%; S2: 7%) skills were gained the least.

**Fig. 2:**
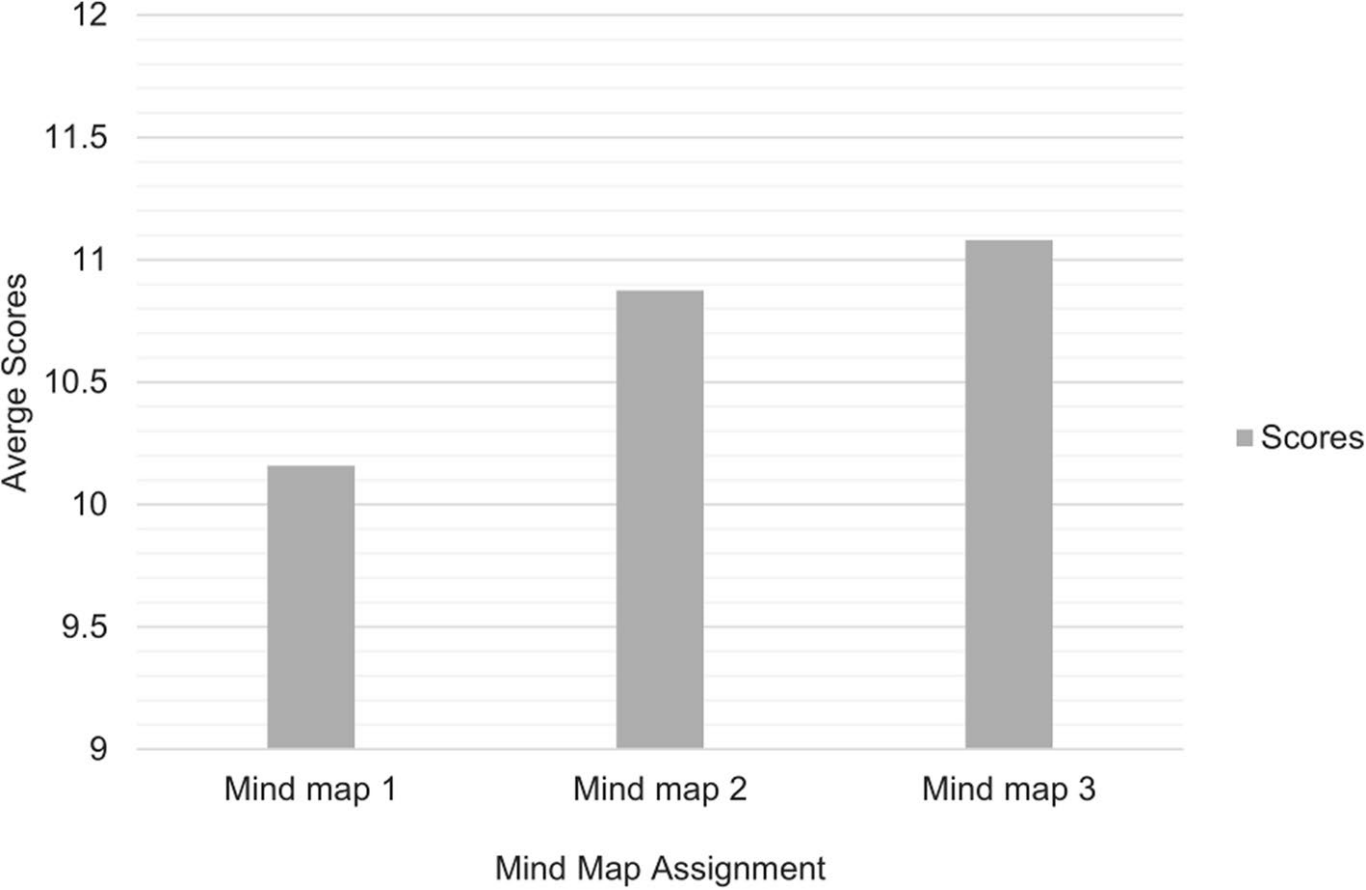
Improvements in students’ average scores out of 12 in both courses with each assignment

In the open-ended question added to S2, students reported the following responses regarding the assignments: 1) positive aspects: they learned new skills, better understood the course, learned in groups, and gained independent learning skills while studying; 2) negatives aspects: shorter submission deadlines, difficulty in group-work for some students, and challenges in identifying key concepts; 3) improvements suggested: allowing students to choose their group members and providing a practice session for students before the first assignment is posted on Blackboard. Figure 2 shows the improvements in students’ results across all three mind maps.

### Students’ satisfaction with mind map assignments and comparison of students’ academic achievement in the current (after COVID-19 pandemic started) and previous term (before COVID-19 pandemic)

The paired samples t-test showed no significant difference between students’ mean satisfaction in S1 and S2. This indicated a lack of post-S2 improvement in students’ satisfaction. For the first objective, a paired samples t-test was performed on students’ average satisfaction in S1 (M = 3.38, SD = 0.85) and S2 (M = 3.53, SD = 0.81), with information regarding mind mapping and associated course assignments. However, no significant differences were found in students’ satisfaction. For the second objective, students’ average responses regarding the effect of changing assessment styles and the transition to online education on students’ satisfaction were compared between S1 ($M=3.06$, SD = 0.92) and S2 (M = $2.98, \mathrm{SD}=0.95$) S2, and the results indicated no significant differences in students’ satisfaction for this objective. Similarly, no significant differences were found for the third objective, between students’ responses regarding satisfaction with using mind maps in S1 $(M =3.74$, SD = 0.95) and S2 $(M=3.56, \mathrm{SD}=0.77)$.

**Fig. 3:**
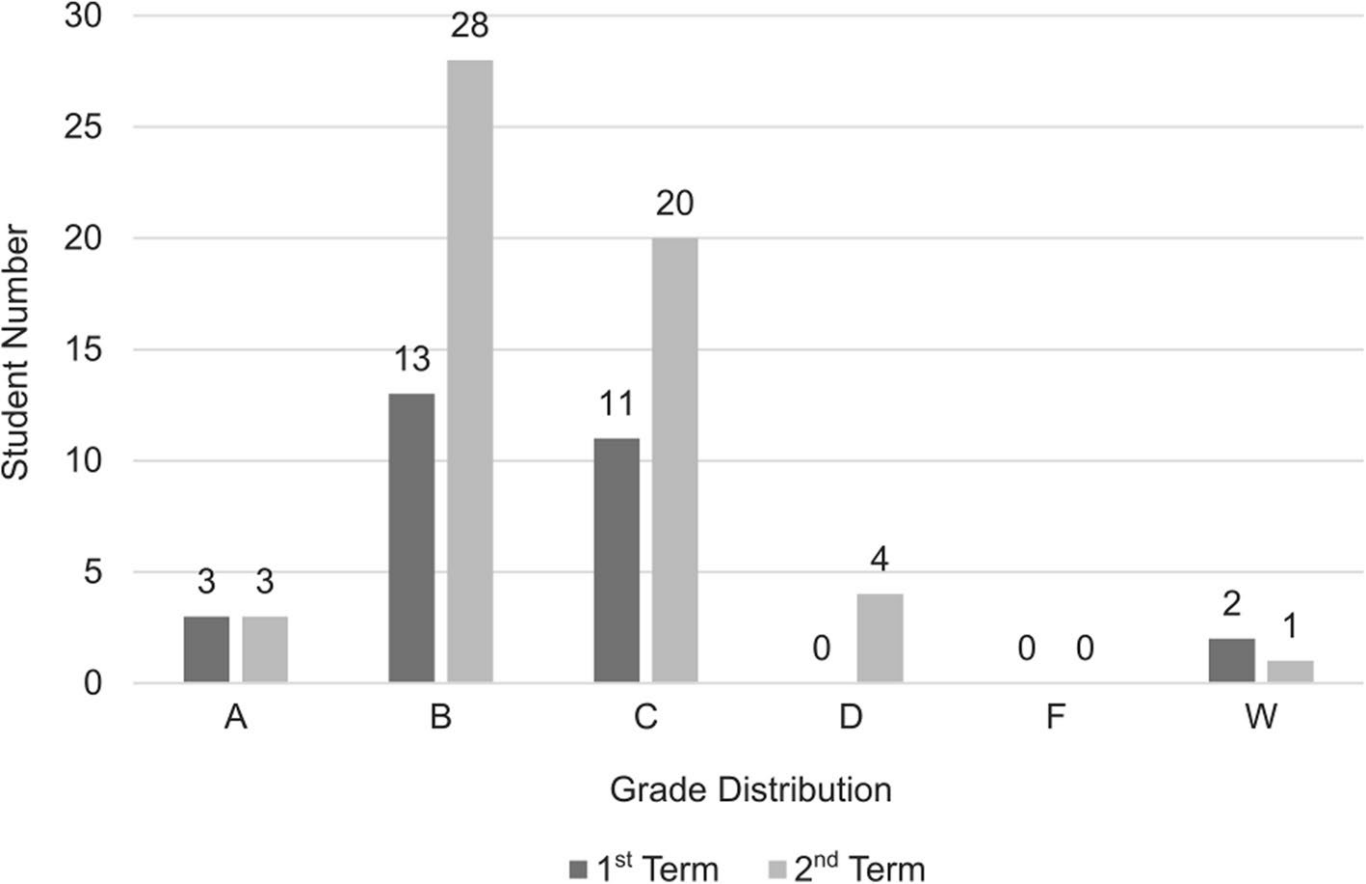
Grade distribution for students in the two courses during the first and second terms in the academic year 2019–2020

Figure 3 shows a normal distribution for students’ grades in the first and second semesters in both the health physics and MRI courses. The result of a chi-squares test shows that there are no differences in students’ grade distribution between the two term of year 2020, before and after COVID-19 pandemic started. Hence, chi-square = 4.29 < ${x}_{\mathrm{0.05,4}}^{2}$  = 9.48 and p-value = 0.36 > 0.05 support hypothesis H_0_, where H_0_ means no relationship between students’ academic achievement and term, where grades are independent of the term, while H_1_ posits there is a relationship between student academic achievement and term, where grades are dependent on the term.

## Discussion

This study investigated the use of mind maps as a learning and assessment tool for medical physics students at KAU under the stress of the COVID-19 pandemic and a sudden switch in teaching mode from on-campus to online classes. Two surveys, one directly after the first mind map assignment and the other just before the final exam, were distributed to students, to assess student satisfaction with the information provided on mind mapping and the associated course assignments, effects of changing assessment styles due to COVID-19 control measures, switching from on-campus classes to online classes, student satisfaction levels, student satisfaction with using mind maps as a learning tool, and the skills gained from the assignments.

The findings showed no significant differences between surveys for students' mean satisfaction scores. This could be due to the sudden changes and control measures that were implemented by the Ministry of Education during the COVID-19 pandemic, as explained above; hence, the study was conducted over a short period, during the second half of the second term of 2020. Moreover, most students had no prior experience with mind maps, and only 19% of the students who conducted the survey (survey S1) said that they learnt and used mind maps before this course. Furthermore, the study was conducted with a limited number of students. However, by comparing the results of students’ responses to each of the surveys’ objectives, we were able to gain a deeper insight. Hence, although students were not initially certain about the usefulness of the information provided, as they completed more assignments, they became more skilled in using mind maps and could make clearer judgments. This confirms that the mind map is a powerful tool that can be easily adapted when suitable guidance is provided, and results can be seen over a short period of time. This is in line with the findings of previous studies [15, 35], which compared memory retrieval and critical-thinking in medical students using mind mapping and standard note-taking techniques. Although there were no differences in the results between the two methods, students did not use mind mapping before participating in the study, which indicates that mind mapping is a robust learning technique.

The mean response was neutral in both surveys regarding the effects of changing assessment styles due to COVID-19 control measures, switching from on-campus to online classes and student satisfaction levels. Students were unable to decide if they agreed or disagreed that the sudden changes were stressful. Moawad [36] studied student responses to academic stress from online learning during the COVID-19 pandemic. The study found that the main source of stress stemmed from uncertainty about exams and assignment changes. These issues were addressed at our university almost immediately after switching to online teaching platforms; there was tremendous support and prompt strategies and polices were provided in response to the pandemic to maintain effective education for students. Student support included short online courses on how to use Blackboard, support for students with special needs, and online academic and psychological counseling, with reassuring messages provided to students on university social media platforms [37].

In both surveys, students were satisfied with the use of mind mapping as a learning tool. This finding confirms that it is an effective learning tool that can improve students’ comprehension and information retrieval skills in medical education [5, 15], enhance learning and vocabulary recall [38], and improve writing in English [22]. Colors, images, and connections are all used in the learning process, which in turn increase students’ motivation to learn [39–41]. Moreover, students’ responses to the open-ended questions in both surveys regarding skills they perceived themselves as acquiring during group work on the mind mapping assignments showed that organizing, planning, and decision-making skills were acquired most frequently, while critical-thinking skills came in third. All these skills are crucial for students’ academic achievement. Abdulghani et al. [16] reported that high-achieving medical students perceived prioritization, time management, and mind mapping as factors that contributed to their academic success. Furthermore, the present study shows that the more the students complete mind map assignments, the more improvement there is in their grades for mind maps. This indicates that continuous application of the mind mapping technique can improve students’ learning, which is reflected in their grades and achievements. Students’ perceptions of the advantages of using mind maps, such as enhanced learning and comprehension, were similar to the results of previous studies by Wickramasinghe et al. [41], Erdem [42], and Wu and Wu [15].

However, some students thought that mind mapping was time consuming and felt that they needed more time, while others thought that identifying the main concept was challenging and there were concerns regarding low grades. These concerns were examined by Erdem [42] in a study focused on university students’ perceptions on the use of mind mapping as a lifelong learning tool, which suggested that as students continue to develop mind maps, they become faster and more skilled, creative, and imaginative. Some students thought that working with mind maps in a group was an opportunity to learn, while others thought it was a disadvantage. While Stokhof et al. [40] concluded that shared mind map activity enhances students’ learning and critical thinking, Jones et al. [39] suggested varying students’ mind mapping activities and allowed students to work on mind maps according to their preferences—individually in or out of class or with groups—could maximize student engagement, thereby improving their learning goals.

Despite the circumstances of the second term in which the COVID-19 pandemic began and education and lifestyle changes were enforced as preventive measures, there are no significant differences in students’ grade distributions between the two terms of the year 2020 (before and after the COVID-19 pandemic began). This is in alignment with a study done by Elsaid [43], whose results showed no significant difference despite this sudden switch. The study also compared students’ grade distribution for the two-learning models in the developing country, taken before the pandemic (face-to-face learning model) and after the COVID-19 pandemic (online learning model). This is also confirmed by studies comparing the performance of online learners with conventional (face-to-face) learners before the pandemic started [44, 45]. Moreover, another study by Gonzalez et al. [46] conducted on Spanish university students showed a significant improvement in students’ performance during the COVID-19 pandemic, despite the teaching model changes. The students believed to be motivated to change their time management habits during studying and learning strategies and improving their independent learning performance to adapt to the uncertainties that came with the pandemic, thus ensuring that their academic advances which back as an improvement in their performances. However, conflicting outcomes were reported in other studies. This is evident in a recent study done by Giusti [47] on Italian students’ perspectives regarding distance learning and its impact on their psychological health and academic performance. The study showed that the negative impact of online education on students is related to psychological factors, such as anxiety and depression. This is due to lack of social interaction, technical, and economic factors. This in turn reflects negatively on students’ performance. The lack of network availability, suitable network coverage and educational platforms, online educational support from courses’ instructors, virtual interaction with colleagues and instructors, efficient online course content, online support and training on the provided online platforms, and financial support for students are also key factors that are reported to affect students’ performance [48, 49]. Adapting a strategic and swift switch to online education could mitigate the dramatic effect that could arise because of sudden and extreme situations, such as the COVID-19 pandemic. For example, to sustain educational support for students, King Abdulaziz University KAU managed to successfully implement an online model where thousands of classes were conducted weekly with the help and support of the Deanship of e-Learning and Distance Education. As a result, AI-Youbi et al. [37] proposed the KAU Pandemic Framework that integrates five pillars of strategic adoption of the social media platform (Twitter): social media governance, social media resilience, social media utilization, decision-making capabilities, and institutional strategies, which help provide flexible and sustainable education and learning overtime. Twitter was used throughout the pandemic time to communicate with the KAU community regarding administrative issues, educational sustainability, and community responsibility. Hence, KAU could communicate all regulations and changes due to the COVID-19 pandemic and at the same time, attend to students needs by providing helplines for student enquires, the supply of free laptops for students in need, online academic advice, psychological support, and online support for disabled students and students with autism. Furthermore, there were many activities organized for community support, such as raising awareness of COVID-19 and safety measures through workshops and infographics.

## Conclusion

The study aimed to measure the effectiveness of mind mapping as a learning and assessment tool for female medical physics student during the pandemic. Hence, students’ satisfaction were also considered by conducting two surveys measuring: the learning material on mind maps and associated course assignments provided, changing the assessment style due to the sudden switch from face-to-face to online learning models, and using mind maps as a learning tool. Students were also asked about their overall experience and skills that they perceived to have gained from the mind map assignments. Although results show no significant difference between students’ mean satisfaction in both surveys, students’ responses showed consistency regarding their satisfaction with using mind maps. The results of the present study indicate that mind-mapping is a powerful learning tool that, if practiced and mastered by students, could enhance their learning outcomes and improve their critical-thinking and soft skills. However, our number of participants was limited. The sample only included two courses that were taught for second- and fourth-year female medical physics students of a Science faculty in KAU. Future studies could include a longitudinal analysis over a longer period. They should also include the effect of some learners’ demographic and academic variations, such as gender, age, degree, and GPA. It is worth expanding the study to include other courses for medical physics within the same institute, and expanding it to compare different subjects within the same faculty, thus improving the scope of the results. Moreover, future collaborative research across institutes could help assess regional differences.

The results also show that the sudden switch in educational settings, from conventional to online due the COVID-19 pandemic, didn’t negatively affect students’ grades. In challenging situations such as these, well-established communication between educational institutions and students is believed to ease students’ stress regarding the effects of changes on their grades and future. For future research, it might be worth involving students in a variety of mind mapping activities—individually in or out of class or with groups—to explore differences in their learning preferences and how this can affect learning experiences.

This study did present certain limitations. First, although students were given the option to choose software programs, some software had limited features, which could cost students money or time when they must learn a new software program. Therefore, to overcome this difficulty, it is recommended that students are provided with one software program that is supported by the educational institution. Second, although students were provided with materials and a short session to explain mind mapping, some students still reporting difficulties in understanding the technique. Thus, it is recommended that students are offered a practice session and a mock mind mapping assignment to ensure that they understand the process fully. To maximize students’ learning experience, it is also suggested that a discussion session be provided, where students can present their work to their peers in class and discuss their ideas and suggestions for improvements. Finally, this study was performed in a limited context; hence, it is recommended that this method be applied to future research using different courses and over a longer period to monitor changes in students’ achievements.

## Supplementary Information

**Additional file 1.**

**Additional file 2.**

**Additional file 3.**

**Additional file 4.**

## Data Availability

The datasets used and/or analyzed during the current study are available as additional files.

## Abbreviations

COVID-19: Coronavirus disease 2019
MRI: Medical imaging
SD: Standard deviations
