# Supplementary material for: The effect of implementing mind maps for online learning and assessment on students during COVID-19 pandemic: a cross sectional study
Source: BMC Med Educ. 2022 Mar 12;22:169. doi: 10.1186/s12909-022-03211-2 (PMC8917332; doi:10.1186/s12909-022-03211-2)
Supplement: Supplementary file 1 — Additional file 1. [file 12909_2022_3211_MOESM1_ESM.docx]

**Additional File 1**. Appendices

**Appendix A.** Mind map assessment rubric

|  | **4** | **3** | **2** | **1** |
| --- | --- | --- | --- | --- |
| **Organization** | The mind map is well organized and in a logical format.  Main concepts are included and branch out logically, with keywords that cover the whole subject. | The mind map is mostly organized and in a logical format. Most main concepts are included and branch out logically most of the time, with keywords that cover most of the subject. | The mind map is somewhat organized and in a logical format.  A few main concepts are included and branch out, with a few keywords that cover part of the subject. | The mind map is confusing and unorganized.  Most main concepts are missing and branch out, with little to no keywords. |
| **Depth and Content** | Uses correct terminology to explain different concepts and principles.  Includes all necessary subtopics and ideas under each subtopic. Enhances deep understanding by adding supportive ideas wherever suitable. | Makes a few errors in the terminology explaining concepts and principles (1 or 2 errors).  Includes at least 4 subtopics and a minimum of 2 ideas under each subtopic.  Enhances deep understanding by adding supportive ideas/information most of the time (2 or 3 examples of supporting information). | Makes many errors regarding the terminology explaining concepts and principles (more than 2 errors).  Includes 3 subtopics and/or is missing the required number of ideas under each subtopic. Somewhat enhances understanding by adding supportive ideas (1 or 2 examples of supporting information). | Shows no understanding of the terminology and related concepts.  Includes only 2 subtopics and/or is missing ideas under each subtopic. No supporting evidence provided to enhance understanding. |
| **Linking Ideas** | Effective use of symbols and arrows to link concepts and different ideas to enhance deep understanding of the subject. | Clear use of symbols and arrows to link ideas and concepts, with a few errors in some connections (1 or 2 errors). | Uses symbols and arrows to link ideas and concepts, with some errors in some connections (more than 2 errors). | Does not convey any meaningful connections between ideas and concepts. |
| **Design and Creativity** | The map layout is interesting and correct. The font is appropriately sized. Colors and images are used correctly and effectively to enhance learning. | The map layout is good and correct.  The font is hard to read in some parts. Colors and images are used correctly and enhance learning in most parts of the mind map. | There are some errors in the layout.  The font is hard to read in most parts. Colors and images are used very infrequently and/or incorrectly. | The layout is incorrect. The font is hard to read. Colors and images are used incorrectly or not at all. |

**Appendix B.** The first student survey

| **Student satisfaction regarding the information provided about mind mapping and associated assignments:**   1. I have learned about and used mind maps in my studies before. 2. The information the instructor provided on the Blackboard about how to create mind maps was very useful to me. 3. The information the instructor provided on the Blackboard about mind mapping software was very useful to me. 4. The information the instructor provided about grading the mind maps was clear.   **Changing the assessment style due to COVID-19 control measures and the transition to online education: Effects on students**  1. Switching course assessment methods, in general, from paper quizzes and tests to online assignments was stressful for me.  2. Switching the assessment method of this course from paper quizzes and tests to online assignments, mind mapping projects, and online MCQ tests was stressful for me.  3. I am satisfied with the changes in grade distribution.  **Student satisfaction with using mind maps as a learning tool and skills gained from the assignment:**  1. Using mind maps was beneficial for my learning and helped me understand the course.  2. Using mind maps helped me identify the main ideas in the lectures.  3. Using mind maps helped me understand the subject in depth.  4. I gained new skills while working in a team for the mind mapping projects.  5. I will continue to use mind maps in my future courses.  **Choose the skills you believe you acquired by completing the course assignments:**  1. Organization and planning skills  2. Decision-making skills  3. Problem-solving skills  4. Persuasion and influencing skills  5. Feedback skills  6.Conflict resolution skills  7. Critical-thinking skills |
| --- |
